# Spatial distribution model of anoa, *Bubalus* spp., in Tanjung Peropa Wildlife Reserve

**DOI:** 10.3897/BDJ.13.e153431

**Published:** 2025-07-30

**Authors:** Ola Prajab Aso, Abdul Haris Mustari, Lilik Budi Prasetyo

**Affiliations:** 1 Tropical Biodiversity Conservation Study Program, Department of Forest Resources Conservation and Ecotourism, Graduate School, IPB University, Bogor, Indonesia Tropical Biodiversity Conservation Study Program, Department of Forest Resources Conservation and Ecotourism, Graduate School, IPB University Bogor Indonesia

**Keywords:** Anoa, distribution, MaxEnt, fruit-feeding trees, conservation

## Abstract

Anoa (*Bubalus* spp.) are an endemic species that inhabits the forests of Sulawesi, but are threatened with extinction due to hunting and habitat destruction. Despite these pressures, they temporally adapt to fragmented lowland forests near rivers with food availability. In the Tanjung Peropa Wildlife Reserve, an isolated habitat patch, we used MaxEnt modelling to assess environmental factors influencing their distribution in support of a comprehensive species conservation programme. We used 233 anoa presence data points from footprints, faeces, food remnants, horn rubbing marks, wallowing areas and shelter locations during field surveys and 13 environmental variables. The MaxEnt model showed excellent performance (AUC 0.923), fruit-feeding trees presence contributing most (41.1%), followed by distance to the road, perimeter metrics, distance to the river, elevation and large patch index metrics. The results show that 1,239.8 ha (3.2%) of the study area is suitable for anoa as primary habitat, 3,045.1 ha (7.8%) is moderately suitable as secondary habitat and 6,609.8 ha (17.0%) is unsuitable out of the total 38,937 ha study area, while the remaining 28,042.3 (72.0%) is undefined due to lack of occurrence data. The anoa distribution map shows that the edge of the area can still provide for the ecological needs of the anoa, highlighting the need for patrols, community education and further surveys of identified and undefined areas.

## Introduction

In recent years, when studying the diversity of mammals in different orders, close attention has been paid to rare endemic species (Gutiérrez and Marinho-Filho 2017, Andreychev and Kuznetsov 2020). The anoa species complex is an endemic group of ungulates found only on Sulawesi and Buton Islands. The expansion of anoa distribution started from the western and central parts of Sulawesi, in line with the expansion of mainland Sulawesi to the emergence of neighbouring islands such as Buton Island (Frantz et al. 2018). Two species within this complex have been classified separately, namely the lowland anoa (*Bubalusdepresicornis*) and the mountain anoa (*Bubalusquarlesi*) (Burton et al. 2005). Based on phylogenetic analysis, mountain anoa are close to lowland anoa (Priyono et al. 2018, Priyono et al. 2020), indicating the evolution of anoa since their ancestors first arrived on mainland Sulawesi in the Pliocene to Pleistocene (Mustari 2019) through Sundaland (Priyono et al. 2024). This biogeographic record makes the anoa related to the Indian buffalo (*Bubalusarnee*) and tamarao (*Bubalusmidorensis*) in the Philippines (Groves 1969).

Human activities are the main threat to anoa survival in the wild (Burton et al. 2005, Lee et al. 2005, Irfan et al. 2018, Rejeki 2018, Arini et al. 2020). Over the past two decades, anoa has been reported as a traded species (Lee et al. 2005), with recent estimates indicating around 283 individuals or 4% of the population, are hunted annually in Sulawesi (Rejeki 2018). The species holds economic and socio-cultural value for some Sulawesi communities, driving illegal hunting and population decline (Lee et al. 2005, Irfan et al. 2018, Rejeki 2018, Arini et al. 2024). Hunting occurs across Sulawesi and Buton, mainly using traps (Lee et al. 2005, Rejeki 2018). Involving local communities in anoa conservation efforts is a crucial step in preventing poaching, by emphasising social, cultural, economic, educational and legal values (Lee et al. 2005, Irfan et al. 2018, Rejeki 2018, Arini et al. 2024). Additionally, habitat fragmentation also isolates populations, increases inbreeding risk and limits gene flow (Mustari 2019, Priyono et al. 2020). Due to the significant pressures, anoa are listed as endangered on the IUCN Red List (IUCN 2023), Appendix I of CITES (UNEP World Conservation Monitoring Centre 2021) and protected under regulation of Ministry of Environment and Forestry Indonesia 2018 No. P.106/2018 concerning Protected Plant and Animal Species.

Anoa is closely tied to forest habitats that provide a variety of vegetation for food, water sources, specific climate, varied topography and areas with low human pressure (Mustari 2009, Wardah et al. 2012, Allo et al. 2018, Ardiani et al. 2023a, Ardiani et al. 2023b, Jaelani et al. 2023). They co-exist with other endemic species such as *Suscelebensis*, *Babyrousacelebensis* and predators like *Pythonreticulatus* (Mustari 2019). Environmental conditions and interspecies interactions influence species distribution (Soberon and Peterson 2005), driven by adaptive responses (Soberon and Peterson 2005, Sinclair et al. 2006) to enhance survival and expand range (Wong and Candolin 2014, Chuang and Peterson 2016), depending on resource availability (Rather et al. 2020). Understanding species distributions has led to new approaches to analysing species relationships with their habitats. One approach that continues to evolve today is species distribution models (SDMs) that integrate species presence or abundance data with environmental conditions at a spatial scale (Elith and Leathwick 2009) with the ability to evaluate ecological variables, identify potential species distributions and project population dynamics to support species conservation management (Merow et al. 2014, Nüchel et al. 2018).

Although various studies have explored anoa distribution at broader spatial scales across Sulawesi using a variety of methodologies (Ardiani et al. 2023a, Ardiani et al. 2023b, Jaelani et al. 2023, Aldiansyah and Wahid 2023), large-scale assessments with limited or no occurrence data may lead to underestimation of distribution in certain habitats. In addition, recent research emphasises that the emergence of localised species highlights the need for small-scale studies to develop more appropriate and site-specific conservation strategies (Dhendup et al. 2024). One habitat of great interest is Tanjung Peropa Wildlife Reserve, which has been identified as having moderate habitat potential (Ardiani et al. 2023a, Ardiani et al. 2023b, Jaelani et al. 2023) and is considered highly vulnerable under the worst-case climate change scenario (Aldiansyah and Wahid 2023). Previous ecological assessments in this area have provided valuable insights into vegetation structure and composition and anoa population densities have been reported to be less than one individual per km² (Mustari 2009, Mustari 2019). In response to this gap in local knowledge and the ecological significance of the area, this study aims to analyse the environmental factors that influence anoa distribution within Tanjung Peropa Wildlife Reserve. We applied the MaxEnt (Maximum Entropy) algorithm, a machine-learning-based species distribution modelling (SDM) approach that effectively handles presence-only data and reduces sampling bias through regularisation of spatial covariates (Elith et al. 2011). The results of this study are expected to directly contribute to the planning of conservation measures and anoa habitat management at the local scale.

## Materials and methods

### Study area

**Figure 1.**
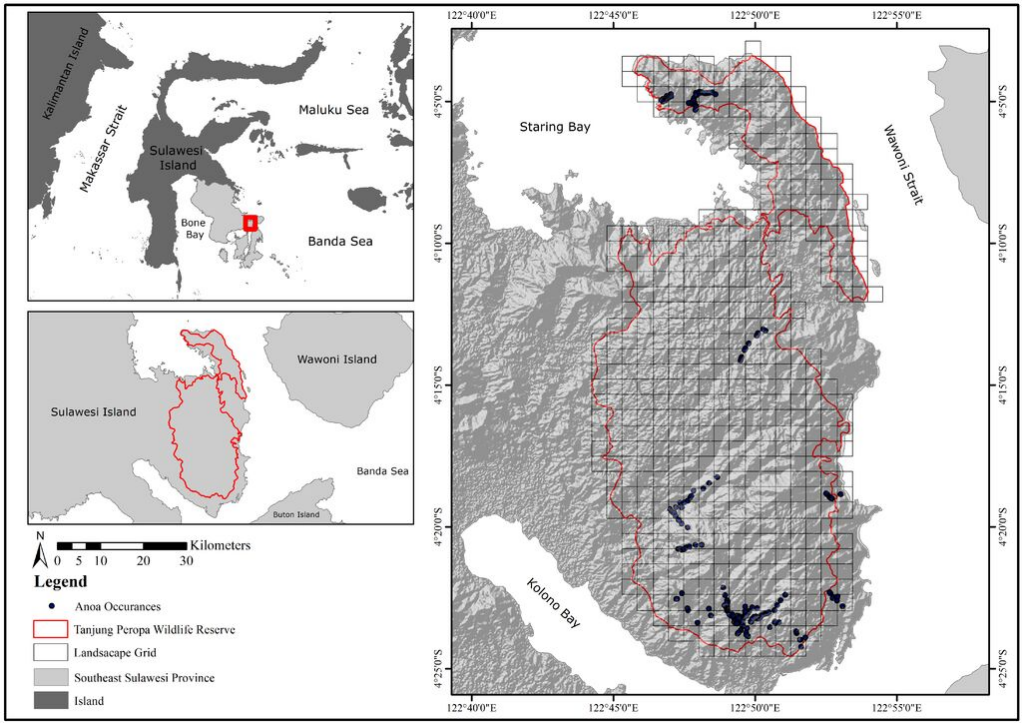
The study area is Tanjung Peropa Wildlife Reserve, southeast Sulawesi, Indonesia.

The study was conducted in Tanjung Peropa Wildlife Reserve (38,937 ha; 4°00'–4°25' N, 122°40'–122°55' E), featuring varied topography up to 934 m a.s.l. and dominated by mixed non-Dipterocarpaceae forest (Fig. 1). This isolated habitat in south-eastern Sulawesi is surrounded by roads, agriculture, settlements and mining. Anoa density is < 1 individual/km², with ~ 350 individuals inhabiting riparian, bamboo and rocky lowland forests above 400 m a.s.l. (Mustari 2009, Mustari 2019). To assess the landscape, we expanded the area to 49,400 ha by adding a 1 × 1 km grid around the Reserve.

### Data collections

Anoa presence data were collected from December 2023 to March 2024 during the dry-to-wet season transition. Surveys involved direct observation of signs (footprints, faeces, food remains, horn rubs, wallows, shelters) along ~ 2 km transects, extended daily from the previous endpoint. A 2–4 person team conducted fieldwork using the cruise method, guided by footprint direction and terrain. One observer consistently measured footprints to reduce bias. All signs were validated with a field guide and geotagged with GPS and photos. Fruit-feeding trees, as potential food sources, were also recorded. A total of 233 presence points were obtained.

Environmental variables were collected from online GIS sources and analysis results, focusing on abiotic, biotic, accessibility (Soberon and Peterson 2005) and anthropogenic factors (Table 1). The distribution data of fruit-feeding trees were obtained from MaxEnt analysis with a range of values from 0-1 as areas with high fruit distribution. Elevation and slope data (30 m resolution) were derived from DEMNAS in Ina-Geoportal (BIG 2018), with slopes calculated used the Slope Tool in ArcMap 10.8. Road and river data came from the same source as the DEM, whereas agricultural land was extracted from land-cover maps and all three processed using Euclidean Distance. The climate data we used were Bioclim data (BIO1: annual temperature) downloaded from global climate and weather data (WorldClim 2020) at 1 km resolution.

Canopy height data were obtained from GLAD (GLAD 2019, Potapov et al. 2021). Landsat 8 imagery (September, 24th 2023) was used to estimate land-cover density using the Forest Canopy Density (FCD) algorithm (Bera et al. 2020), producing five classes (very low to very high), analysed via Google Earth Engine. Sentinel-2 data (Jan–Dec 2024) were classified using Object-Based Image Analysis (OBIA) and the random forest algorithm (Sukristiyanti et al. 2021, Purwanto et al. 2022) on the same platform. Land cover classes included: 1 (water), 2 (open land), 3 (settlement), 4 (agriculture), 5 (shrubs) and 6 (forest), with an overall accuracy of 84%. The forest class was extracted for landscape analysis in Tanjung Peropa. We used the landscapemetrics package (Hesselbarth et al. 2019) in R v.4.4.2 (R Core Team 2024) to calculate 13 landscape metrics (McGarigal and Marks 1995). All data were standardised to UTM WGS 1984 Zone 51S, with a spatial resolution of 30 m.

### Data Analysis

Overlay analysis was performed to examine the environmental characteristics of anoa encounter points, used terra (Hijmans 2025), sp (Pebesma and Bivand 2005), dyplr (Wickham et al. 2025) and ggplot2 (Wickham 2016) in R v.4.4.2 (R Core Team 2024). Each ecological variable was categorised into classes and encounter points were analysed, based on the percentage presence within those classes (Putri et al. 2020). Prior to model development, a multicollinearity test was conducted on 20 environmental variables using Pearson correlation. Variables with r ≥ 0.70 were excluded to avoid redundancy and reduce potential biases that negatively impact model results (Pearson et al. 2006, Dormann et al. 2012, De Marco and Nóbrega 2018). Correlation matrices were calculated in R v.4.4.2 (R Core Team 2024) using the terra package (Hijmans 2025) and visualised with corrplot (Wei and Simko 2024), while scatterplots were generated used ggplot2 (Wickham 2016).

SDM was conducted using MaxEnt 3.4.4, requiring only species and environmental data as predictors. We applied 30% random test (Phillips et al. 2006), 15 replications, 5000 iterations, regularisation multiplier 1, auto features (linear, quadratic, product, hinge), convergence threshold 0.00001, logistic output and subsample replication (Phillips and Dudík 2008, Elith et al. 2011). Variable contribution and permutation importance were averaged across replications to identify key predictors. Model performance was evaluated using threshold-independent ROC (AUC), where values approaching one indicate higher accuracy (Phillips and Dudík 2008, Mousazade et al. 2019, Li et al. 2020). Variable influence was assessed via percent contribution, permutation importance and jackknife test (Phillips et al. 2006, Phillips and Dudík 2008). MaxEnt outputs probability values were 0–1. We mapped predicted anoa distribution in ArcMap 10.8 using classification natural breaks. This method was chosen for its highest Goodness of Variance Fit across ACRC and non-ACRC settings. It effectively reflects the natural distribution pattern of spatial data by minimising within-class variance and maximising between-class variance (Crisana 2014). Given the uneven ecological distribution of habitat suitability, its ability to handle ecological heterogeneity makes it well-suited for conservation-focused classification. Our suitability classes divide into four categories: suitable, moderately suitable, unsuitable and undefined (Boitani et al. 2007).

## Results

### Identification the distribution of the anoa in Tanjung Peropa Wildlife Reserve

**Figure 2.**
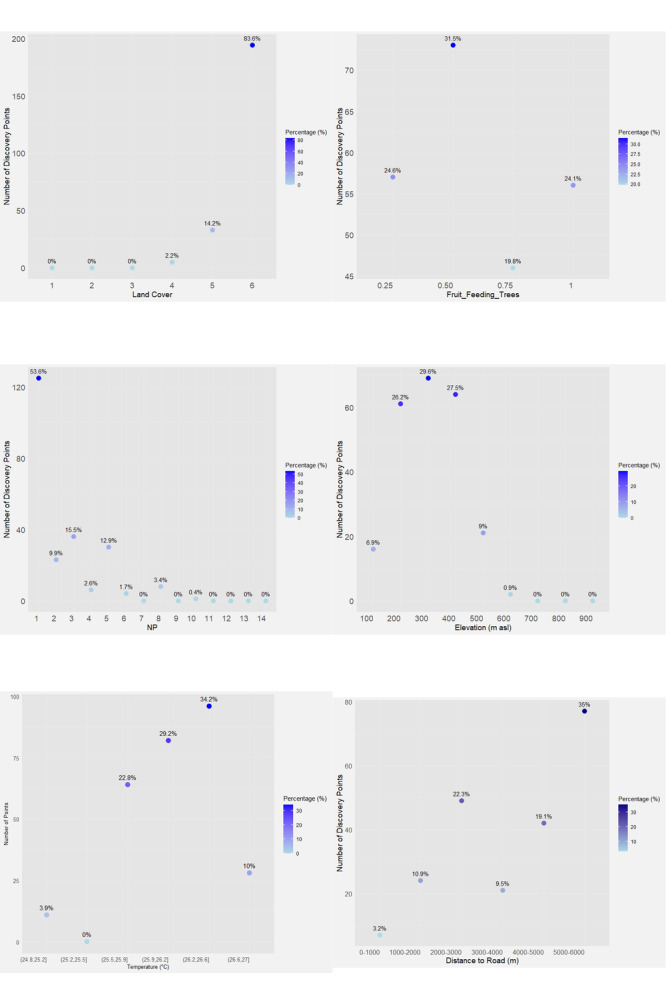
The relationship between each variable and the number of anoa discovery points.

Anoa were predominantly encountered in forest cover (83% of 233 points), characterised by dense canopies (60%–100%) and heights > 20 m, indicating a preference for tall, closed forests (Fig. 2 and Suppl. material 1). Landscape metrics showed occurrences in large and contiguous patches. Based on NP metrics, sightings were concentrated in areas with a single dominant patch, with LPI values exceeding 75%. Additionally, 35.5% of MPS ranged from 76.1–94.8 ha, with LSI of 1–2.5 and MSI of 2, suggesting relatively large and simply-shaped habitat patches.

Based on Class Area (CA) metrics, 50.7% of anoa encounters occurred in patch sizes of 77.6–95.5 ha. Over 30% were found in patches of 76.1–94.8 ha, with perimeters of 2,292.5–3,910 m, shape indices of 1.35–2.06 and core areas of 67.4–84.2 ha, indicating a preference for large, intact forests with significant core areas (Suppl. material 1). Encounters also overlapped with moderate forage tree probabilities (~ 0.5), accounting for 31.5% of sightings (Fig. 2 and Suppl. material 1), followed by 0.25 (24.6%) and 1.0 (24.1%), with the lowest at 0.75 (19.8%). These results suggest anoa select habitats based on the consistent availability of leaves and shoots, their primary food source.

Most encounters (83.3%) occurred below 500 m a.s.l., especially at 200 m (26.2%), 300 m (29.6%), and 400 m (27.5%) (Fig. 2 and Suppl. material 1). They were mainly found on gentle slopes: 0–8° (33.9%) and 8–15° (35.2%). Anoa also preferred areas with annual temperatures of 25.5–26.6°C (86.2%). Regarding anthropogenic pressure, the highest encounter rate (35%) was at 5,000–6,000 m from roads and lowest (22.3%) at 2,000–3,000 m, suggesting avoidance of road-adjacent areas. Encounters were most common 2,000–2,500 m from agriculture (31%), but still occurred within 0–500 m (15.9%) (Fig. 2 and Suppl. material 1).

### Influence Value Between Environmental Factors

**Figure 3.**
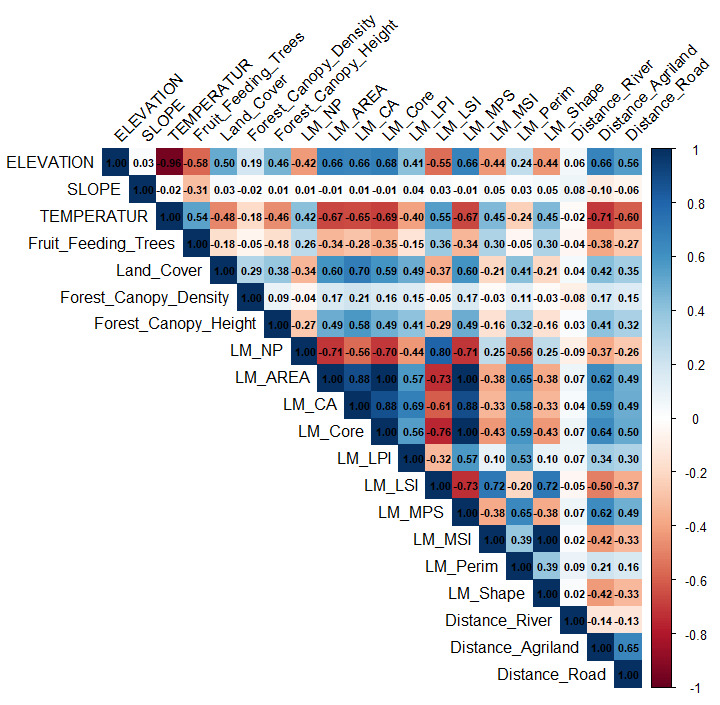
Multicollinearity Analysis (Notes: red for positive, blue for negative correlations).

The correlation heatmap (Fig. 3) reveals strong correlations amongst several landscape metrics, indicating potential redundancy and multicollinearity that could affect model accuracy. AREA strongly correlates with NP, CA, Core, LSI and MPS, while CA is linked to land cover, Core and MPS. Core shows negative correlations with NP and LSI, but positive with MPS, suggesting that larger core areas are associated with fewer, but larger patches. LSI correlates with NP, MPS, MSI and Shape, reflecting the influence of shape complexity on patch characteristics. MPS is also closely related to NP, Shape and MSI. Additionally, temperature shows strong negative correlations with elevation and distance to agriculture, indicating cooler conditions at higher elevations and further from farmland. Due to these strong correlations, variable selection was based on reducing multicollinearity. As a result, temperature and six highly correlated landscape metrics were excluded and the final model retained only variables with low to moderate correlation to improve predictive accuracy.

### Distribution model of anoa

The results of the analysis show that the distribution of fruit-feeding trees (41.1%), distance to road (18.7%), perimeter (9.4%), distance to river (6.4%), elevation (5.5%) and LPI (5.0%) are the seven predictor variables that have importance greater than 5% in running the model (Table 2). Fruit-feeding trees and distance to road are the most dominant factors in determining anoa distribution, based on percentage contribution and permutation importance. Variables such as perimeter, distance to the river and elevation also have significant contributions. In contrast, variables, such as land cover, forest canopy density, slope and forest canopy height, have very little influence on the model and are most likely not decisive in the prediction results.

The contribution of each variable was assessed using jackknife tests for training gain, AUC and test gain (Fig. 4). Variables such as distance to agriculture, distance to road, elevation, MSI, perimeter and fruit-feeding trees consistently showed the highest contributions across all metrics, confirming their key role in predicting anoa habitat distribution. Amongst them, fruit-feeding trees had the strongest impact and removing it significantly reduced model performance. Excluding any of these six variables led to a noticeable drop in AUC, highlighting their irreplaceability. In contrast, variables like land cover, forest canopy density and canopy height showed low contribution and removing them improved model performance. Variables with moderate to low contribution, including distance to river, LPI and NP, also influenced the model, though their exclusion caused minimal change in AUC.

**Figure 5.**
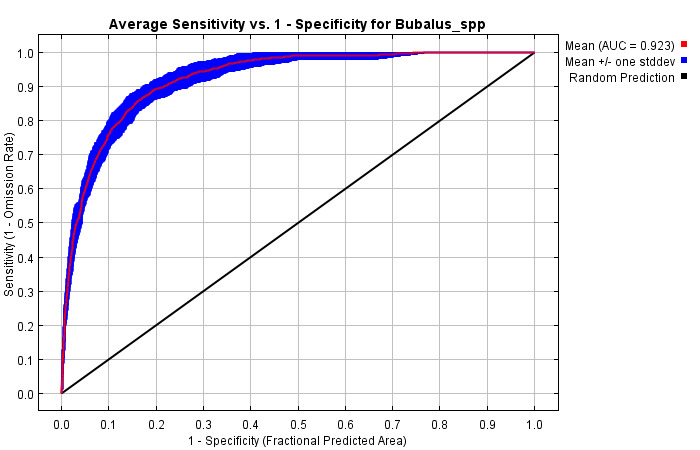
Illustration of the AUC value, based on the receiver operating characteristic (ROC) curve.

SDM of anoa in Tanjung Peropa SM using MaxEnt has a very high accuracy value, based on the Area under Curve (AUC) value on the ROC curve of 0.923 (Fig. 5) and the standard deviation is 0.011. Using a 30% subsample and an average of more than 15 replicates revealed an excellent predictive ability to differentiate anoa distribution across the study area. For example, the model can be reliably used in anoa habitat conservation and management to identify protection areas or expand conservation areas.

**Figure 6.**
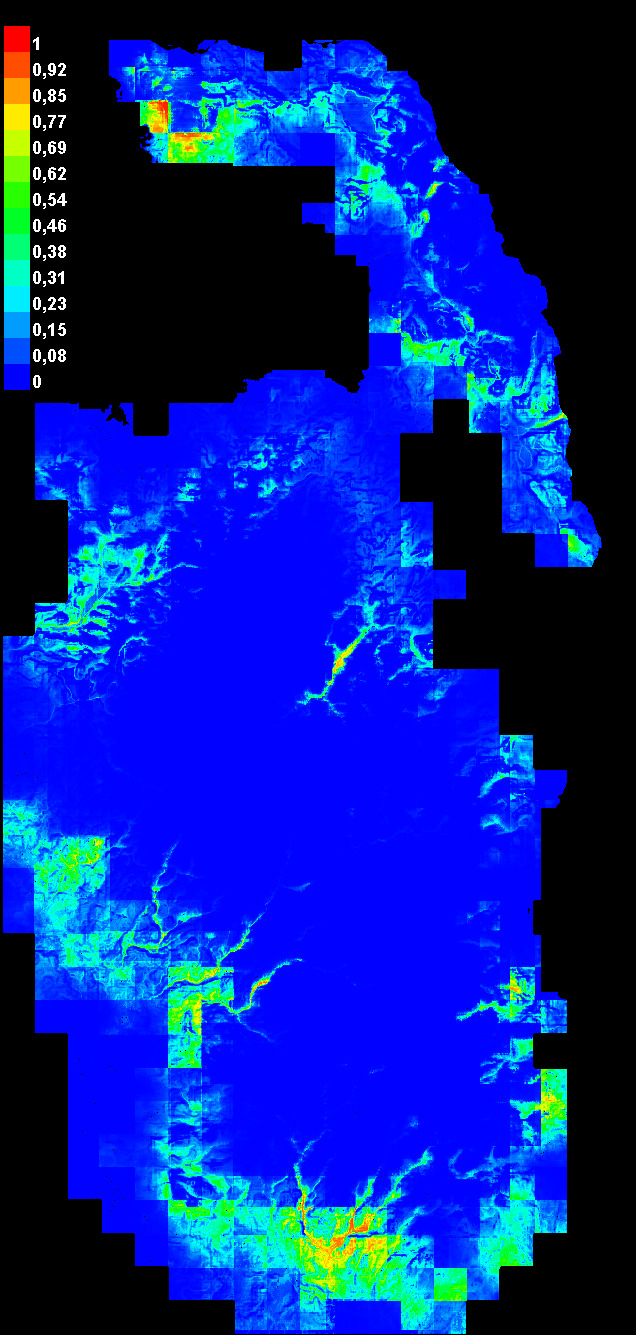
Continuous map from the MaxEnt algorithm with red indicating highly suitable areas and blue indicating unsuitable areas.

**Figure 7.**
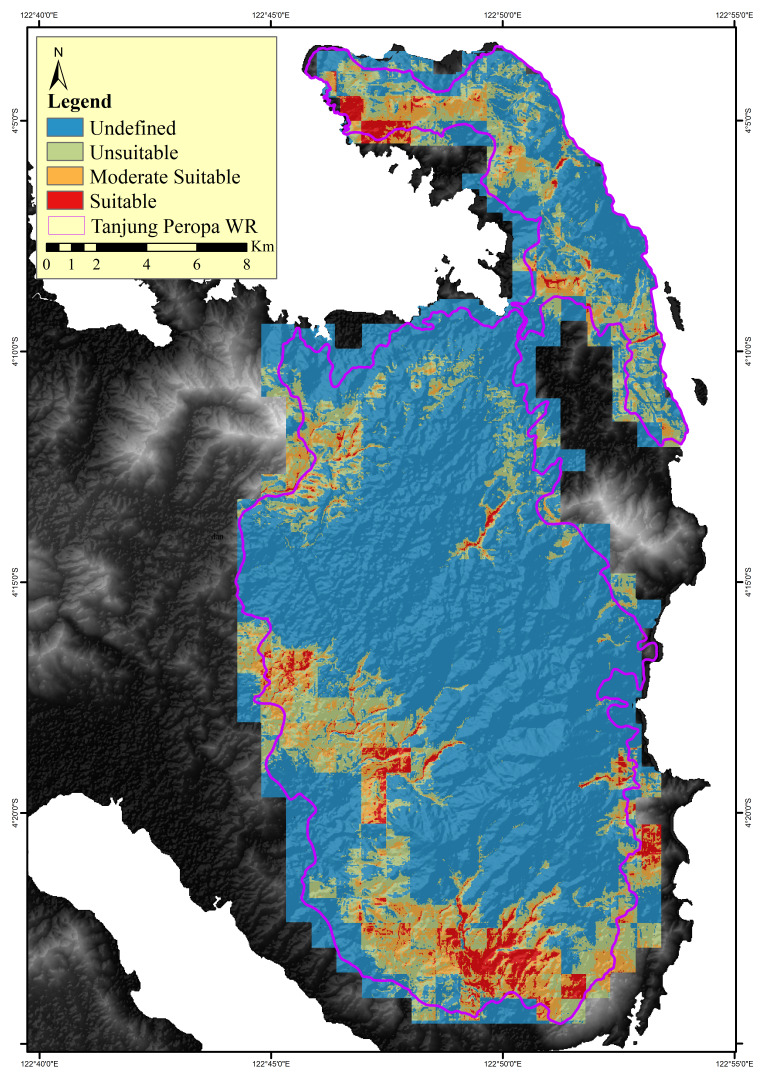
A classification map of anoa distribution.

The distribution of anoa in Tanjung Peropa Wildlife Reserve was modelled using 233 presence points and 11 environmental variables in MaxEnt, resulting in a habitat map (Fig. 6). Habitat suitability was classified into four classes: suitable, moderately suitable, unsuitable and undefined (Fig. 7). Within the Reserve, 3.2% (1,239.8 ha) was suitable, 7.8% (3,045.1 ha) moderately suitable, 17.0% (6,609.8 ha) unsuitable and 72.0% (28,042.3 ha) undefined (Table 3). Outside the Reserve, these classes covered 228.9 ha, 745.7 ha, 1,908.9 ha and 7,779.6 ha, respectively.

## Discussion

Anoa in Tanjung Peropa Wildlife Reserve show adaptations that allow them to roam both forest and non-forest habitats, though their distribution remains highly dependent on forest presence. While previous studies reported anoa occurrences in remote, high-elevation forests (> 1,000 m a.s.l.) with cool temperatures (< 22°C) and varying slopes (Wardah et al. 2012, Allo et al. 2018, Ardiani et al. 2023a, Ardiani et al. 2023b, Jaelani et al. 2023), our findings indicate that landscape characteristics also influence distribution. Anoa were frequently encountered in lowland and riparian forests below 500 m a.s.l., with annual temperatures of 23–26°C. These habitats are characterised by high canopy density and height, supporting diverse vegetation from tree layers to understorey plants, dominated by seedlings and saplings (Purnama et al. 2019). Analysis of 10 landscape metrics shows that anoa prefer landscapes with moderate complexity and low fragmentation, favouring large, intact forest patches as core areas. Although fragmentation can reduce biodiversity and resource availability (Prasetyo 2017), anoa encounters in fragmented areas suggest the presence of critical resources. These areas are often near agricultural land (< 500 m) and ~ 2 km from roads, where fruit-bearing species like *Ficusvariegata*, *Artocarpusdasiphyllus*, *Ficus* spp., *Dilleniaserrata* and *Syzygium* sp. are found. The ongoing fruiting of Ficusvariegata and Dilleniaserrata likely supports anoa activity in these zones. Resource availability, especially food, appears to drive temporal adaptation, allowing anoa to explore areas closer to human presence.

The model developed using 13 environmental variables identified fruit-feeding trees as the primary factor influencing anoa distribution. A total of 41 fruit species from 16 plant families including Moraceae, Myrtaceae, Dilleniaceae and Sapindaceae were recorded as part of the anoa diet in Tanjung Peropa Wildlife Reserve. Ecologically, anoa are browsers, not frugivores, differing from other Bubalus species that are grazers (Sianga et al. 2017, Mandinyenya et al. 2020, Subedi et al. 2023). Previous studies have reported fruit-consuming anoa diets ranging from 10–17% (Arini and Wahyuni 2016, Mustari 2019), typically from fallen fruits or low-hanging species like Ficus and Syzygium. These fruits are a key energy source supporting metabolic needs and movement across varied terrain Mustari (2019). Distribution analysis showed the highest encounter rates in areas with moderate fruit-feeding tree availability, indicating that, while fruits are important, anoa also rely on year-round resources like leaves and shoots. This dietary flexibility reflects a strong ecological interaction between anoa and their habitat, where they contribute to forest regeneration and structure (Butt et al. 2015, Albrecht et al. 2015). However, the distribution of fruit-feeding trees is shaped by topography and climate (Chardon et al. 2019, Khwarahm 2020, Ahmadi et al. 2023), suggesting that climatic factors may indirectly influence anoa distribution and survival.

Identifying environmental characteristics in areas utilised by anoa through species distribution models (SDMs) is a crucial initial step in conservation planning. The distribution map (Fig. 6 and Fig. 7) highlights three habitat suitability classes, with suitable habitats primarily located along the Reserve’s edges. The SDM performed well, indicating high model reliability and supporting its use in guiding conservation priorities (Thorn et al. 2009, Franklin 2013, Kufa et al. 2022). Although anoa presence data from the core zone were lacking, track observations suggest movement from the core to edge and surrounding areas. Approximately 70% of the landscape remains classified as "undefined" likely due to limited survey coverage. According to Boitani et al. (2007) and Feng et al. (2019), such areas may represent unsuitable, secondary or even suitable habitats with low detectability. The concentration of suitable habitats at the periphery indicates the continued availability of key resources and underscores the need for further research to provide information for conservation strategies.

Overall, anoa presence in Tanjung Peropa Wildlife Reserve is strongly associated with large forest patches. However, this study also recorded occurrences in slightly fragmented areas near human activity. While habitat fragmentation generally reduces species presence (Estrada et al. 2017, Kufa et al. 2022), the landscape connectivity between forest and non-forest areas in this Reserve appears to support anoa movement and resource use through temporal adaptation. Monitoring resource availability in both forested and adjacent non-forested habitats is essential to better understand anoa presence and habitat use. For effective conservation planning, further data are needed on population density and habitat carrying capacity in suitable areas. In addition, human activities, such as poaching and illegal logging, must be monitored to prevent potential conflicts. Given that 72% of the Reserve is still classified as undefined, further surveys are necessary to clarify the status of anoa populations in these areas and how they utilise habitats at the core and edges of the Reserve.

## Conclusions

Understanding species ecology is fundamental to guiding effective conservation efforts. The MaxEnt model developed in this study highlights the importance of fruit-feeding trees and flat lowland forests near rivers in shaping anoa distribution. Although human-related variables did not show strong negative effects in the model, anoa presence is influenced by proximity and temporal use of areas near human activity. The concentration of suitable habitats at the Reserve’s edges, within fragmented landscapes, underscores the need for stricter monitoring and targeted conservation. We recommend conservation efforts focused on estimating the optimal density of key food-providing tree species to support habitat sustainability and carrying capacity within and around Tanjung Peropa Wildlife Reserve. Comprehensive assessments of anoa population size, density and connectivity across suitable habitats are also essential. Conservation actions should include habitat restoration, anti-poaching patrols starting from high-suitability edge areas and community education to foster local participation in protecting anoa populations.

## Supplementary Material

AF009FC9-1E92-5F3C-8516-B21B97607375 10.3897/BDJ.13.e153431.suppl1 Supplementary material 1 Identification of the Distribution of Anoa Data type Image Brief description Identification of the Distribution of Anoa with Ecological Variables in Tanjung Peropa Wildlife Reserve. File: oo_1252868.docx https://binary.pensoft.net/file/1252868 Ola Prajab Aso, Abdul Haris Mustari, Lilik Budi Prasetyo

## Figures and Tables

**Figure 4a.**
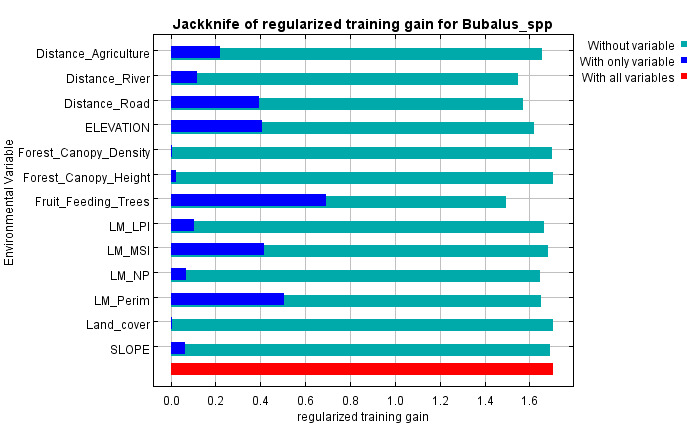
Training

**Figure 4b.**
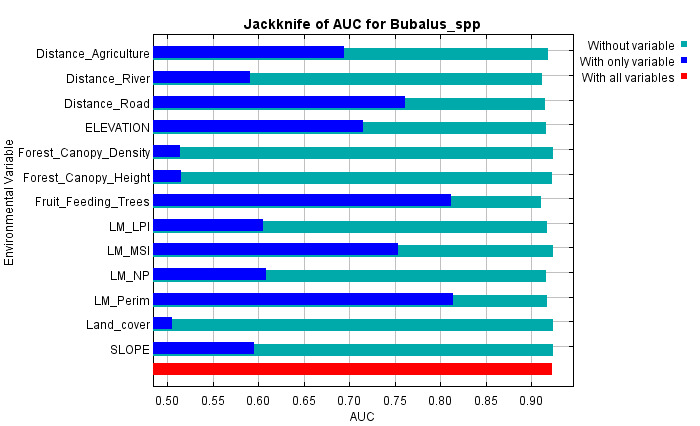
AUC

**Figure 4c.**
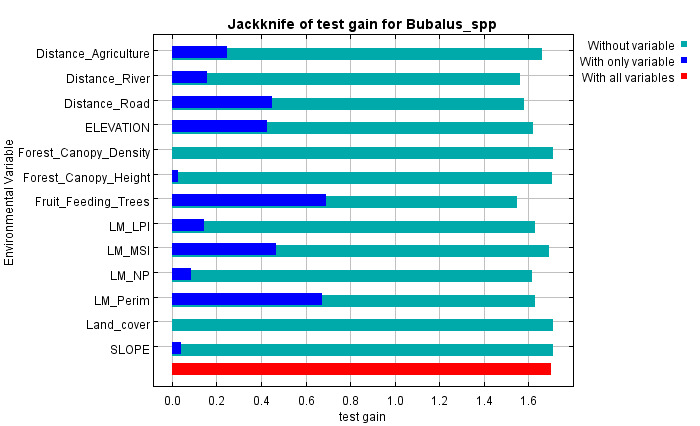
Test

**Table 1.** Ecological predictor variables selected for spatial distribution model anoa.

Type	Variables	Units	Data Sources
Abiotic	Elevation	Metres above sea level	http://tanahair.indonesia.go.id
	Slope	°degree	http://tanahair.indonesia.go.id
	Annual temperature (BIO1)	°C	www.worldclim.org/bioclim
	Distance to river	Metre	http://tanahair.indonesia.go.id
Biotic	Fruit-feeding trees	-	MaxEnt 3.4.4 Analysis
Accessibility	Land cover	-	Google Earth Engine
	Forest canopy density	%	Google Earth Engine
	Forest canopy height	Metre	https://glad.umd.edu/dataset/gedi
	Area (AREA)	Hectare	Google Earth Engine
	Class area (CA)	Hectare	Google Earth Engine
	Large patch index (LPI)	%	Google Earth Engine
	Mean patch index (MPS)	Hectare	Google Earth Engine
	Number of patch (NP)	-	Google Earth Engine
	Perimeter (PERIM)	Metre	Google Earth Engine
	Shape index (SHAPE)	-	Google Earth Engine
	Large shape index (LSI)	-	Google Earth Engine
	Mean shape index (MSI)	-	Google Earth Engine
	Core area (CORE)	Hectare	Google Earth Engine
Anthropogenic	Distance to road	Metre	http://tanahair.indonesia.go.id
	Distance to land agriculture	Metre	Google Earth Engine

**Table 2.** Analysis of Variable Contribution

**Predictor Variable**	**Percentage contribution**	**Permutation importance**
Fruit-feeding trees	41.1	31.2
Distance road	18.7	17.9
Perim	9.4	13.2
Distance river	6.4	8.9
Elevation	5.5	5.4
LPI	5.0	3.3
MSI	4.3	6.4
NP	3.2	7
Distance to agriculture	2.4	5.4
Land cover	1.4	0.1
Forest canopy density	1.2	0
Slope	0.9	0.6
Forest canopy height	0.5	0.7

**Table 3.** Suitability class of anoa distribution in Tanjung Peropa Wildlife Reserve.

No	Class	Area (Ha)	Percentage (%)
1	Undefined	28,042.3	72.0%
2	Unsuitable	6,609.8	17.0%
3	Moderate suitable	3,045.1	7.8%
4	Suitable	1,239.8	3.2%
