# Supplementary material for: Spatial distribution model of anoa, Bubalus spp., in Tanjung Peropa Wildlife Reserve
Source: Biodivers Data J. 2025 Jul 30;13:e153431. doi: 10.3897/BDJ.13.e153431 (PMC12329418; doi:10.3897/BDJ.13.e153431)

**Supplementary file 1: Identification of The Distribution of Anoa with Ecological Variables in Tanjung Peropa Wildlife Reserves.** Overlay results between anoa encounter points and ecological variables in Tanjung Peropa Wildlife Reserves.


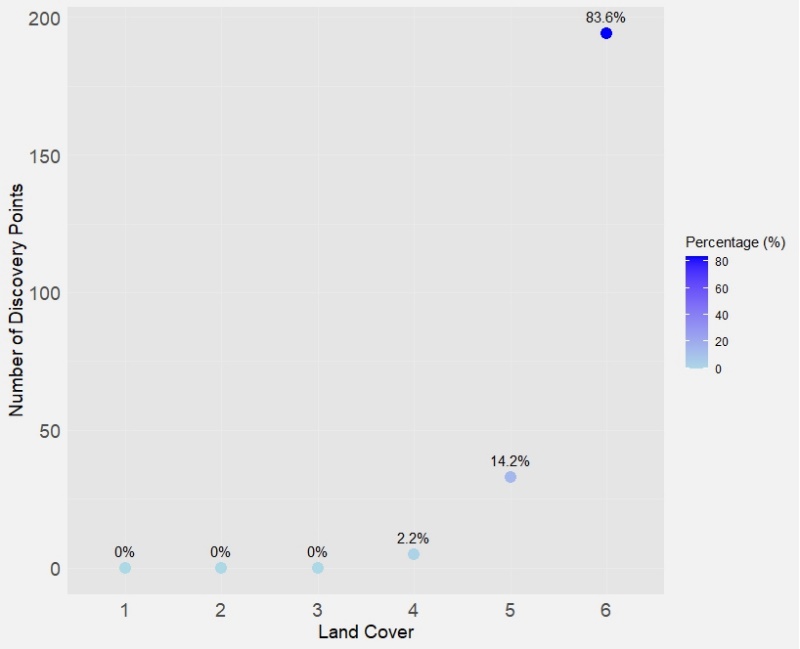

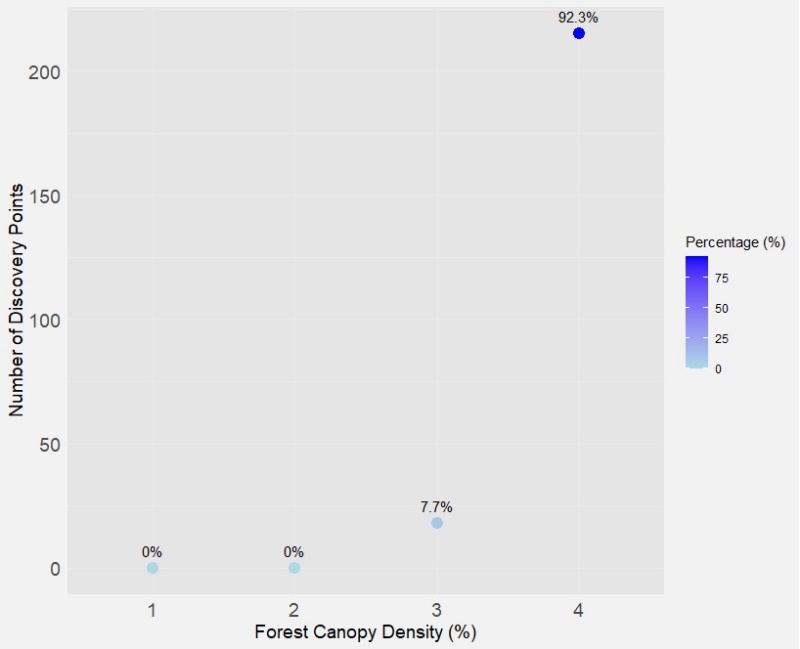

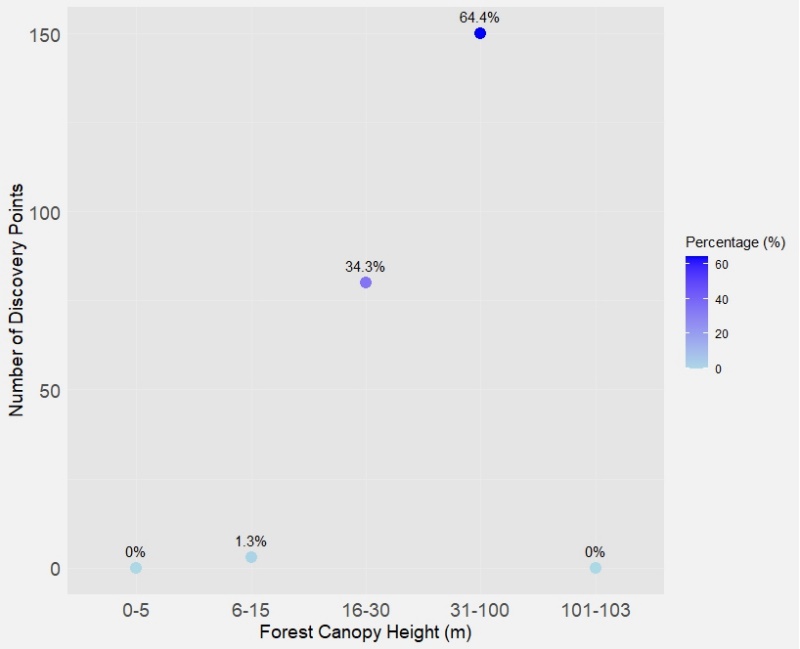

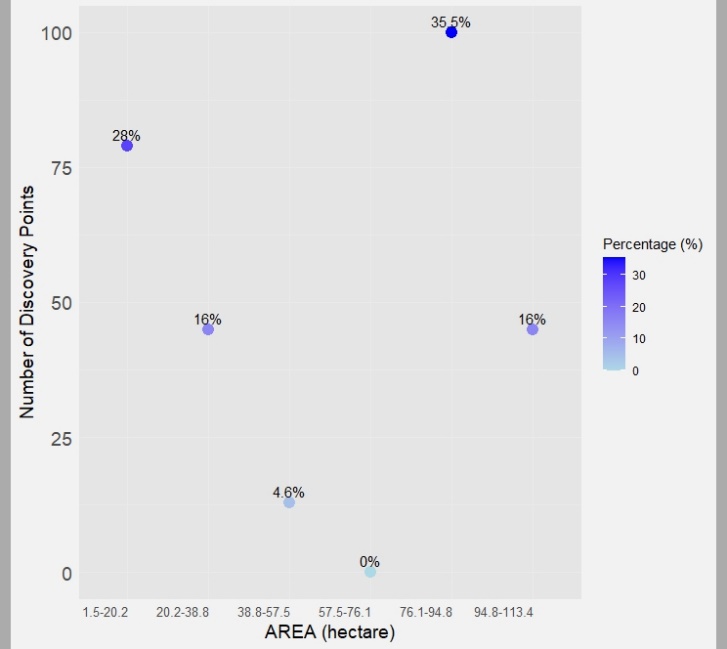

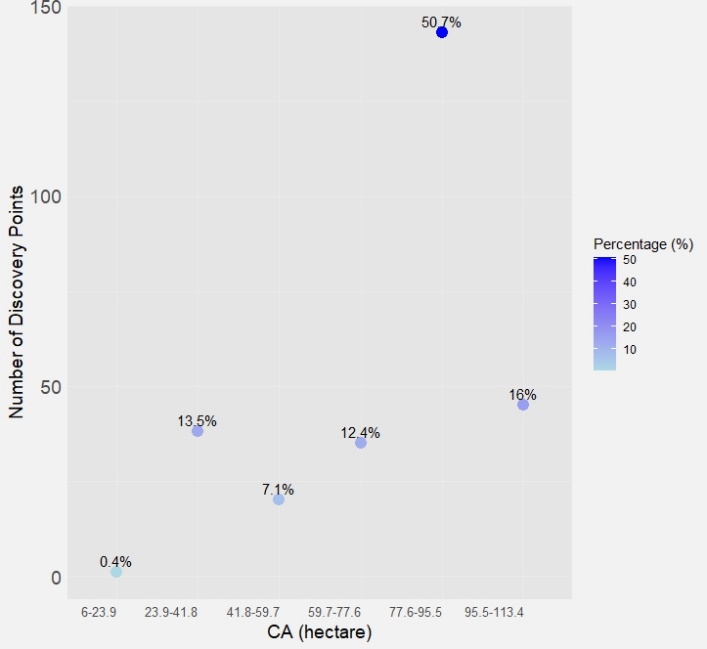

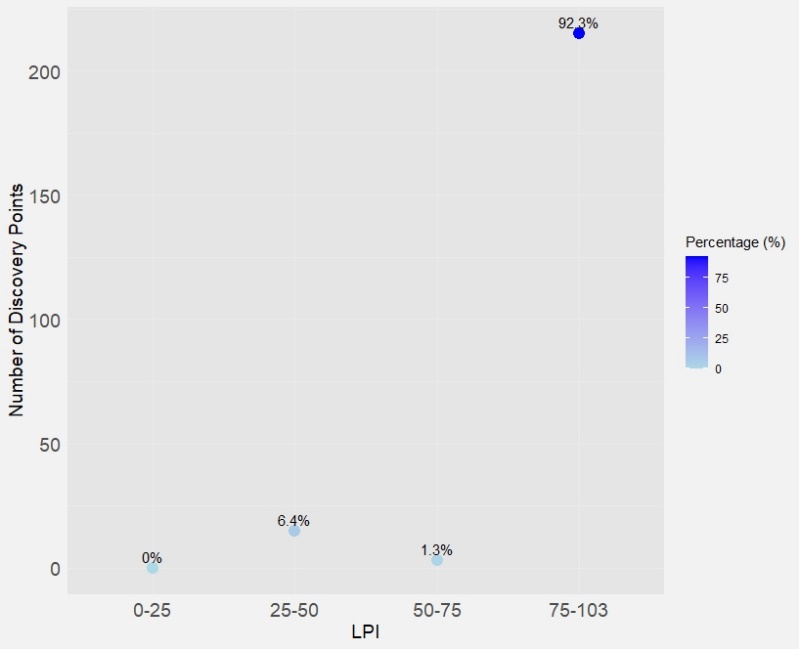

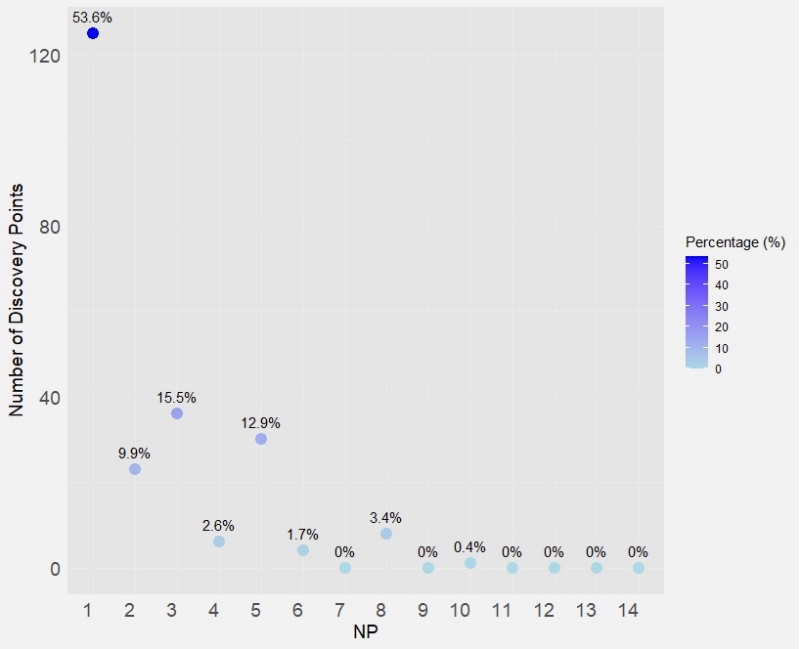

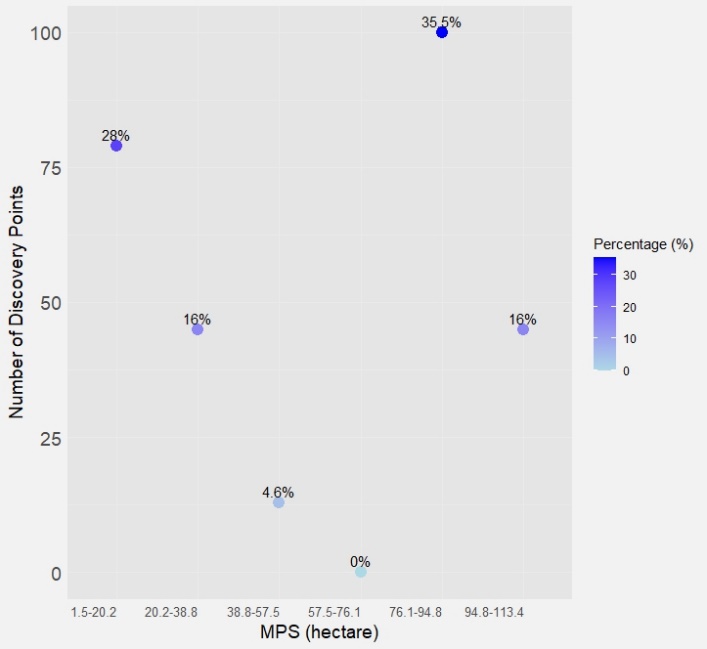

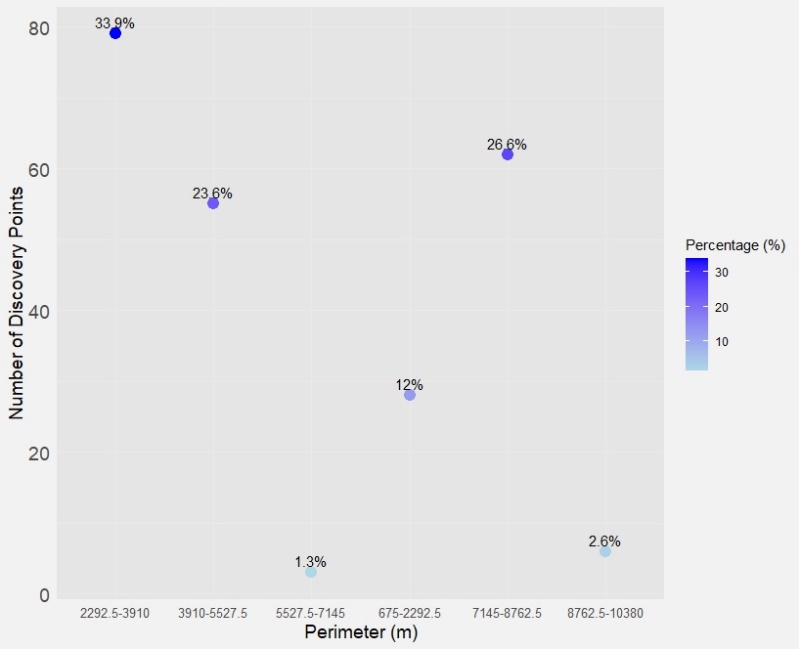

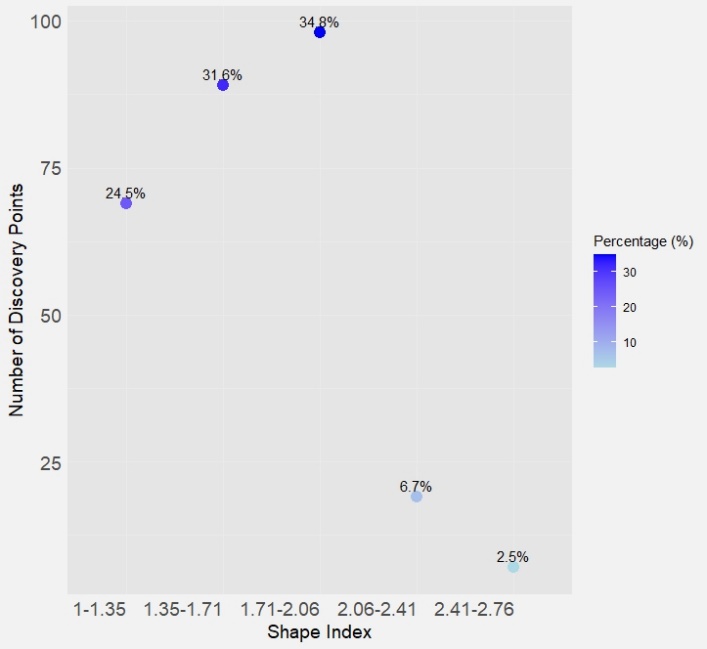

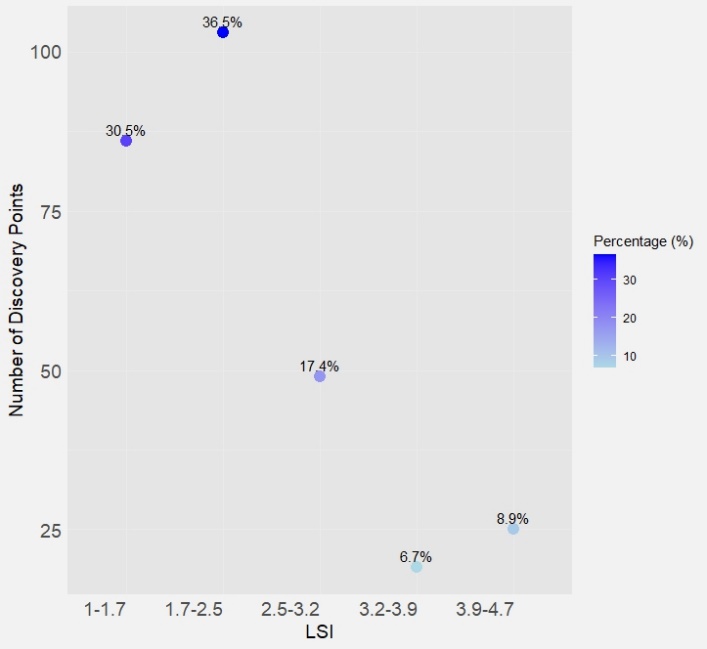

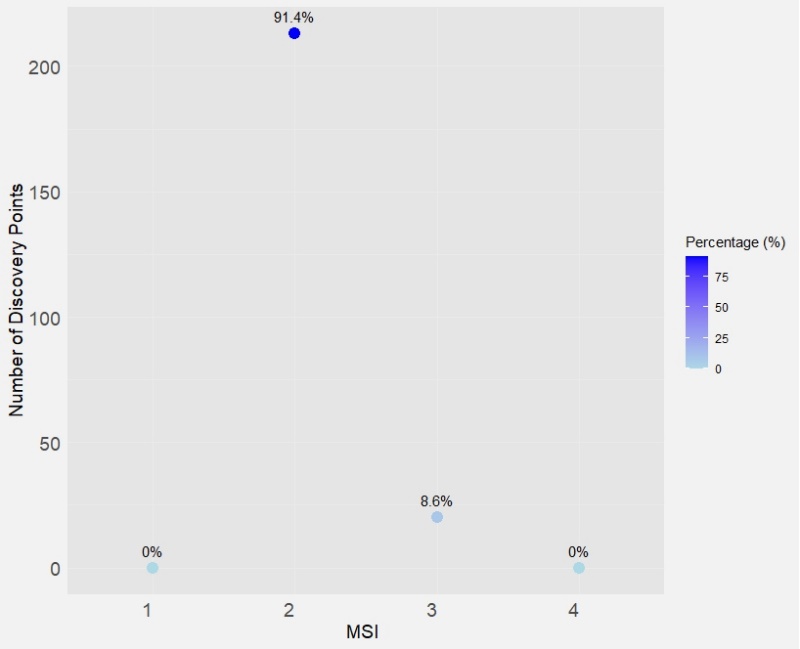

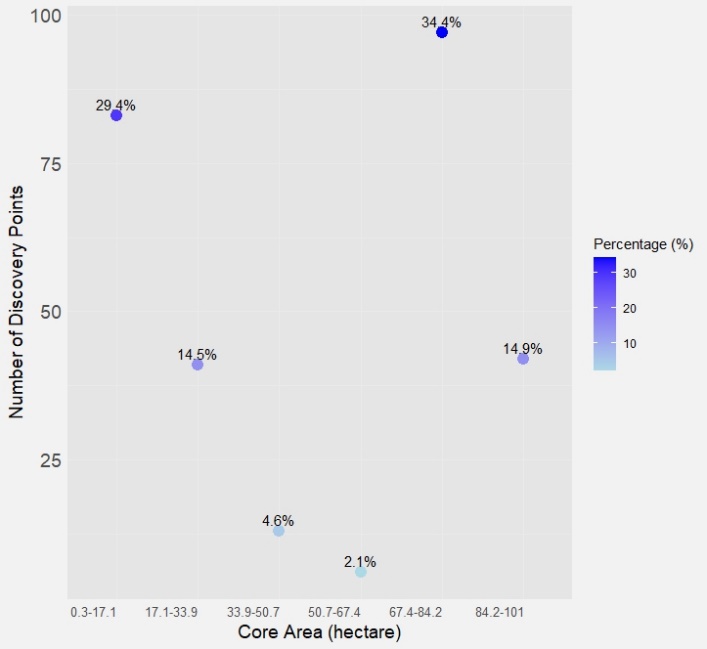

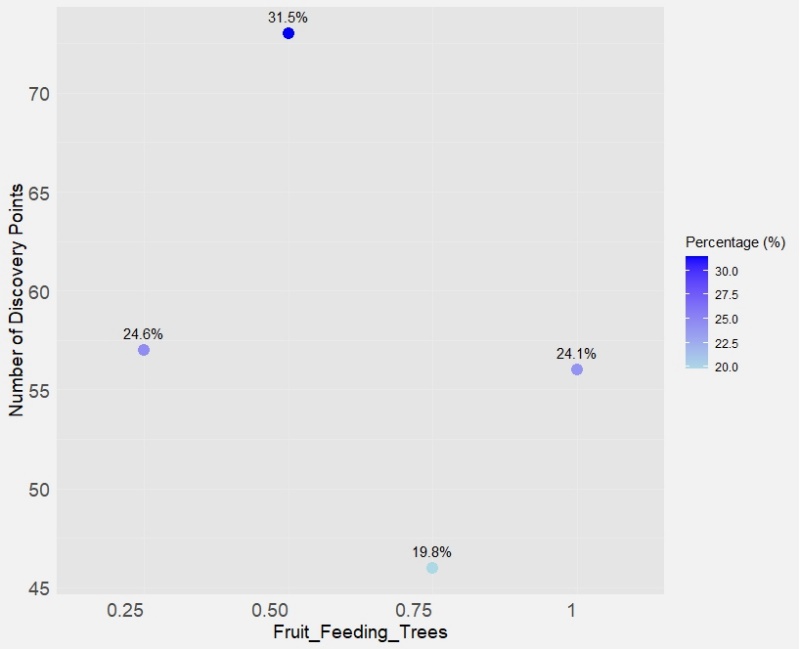

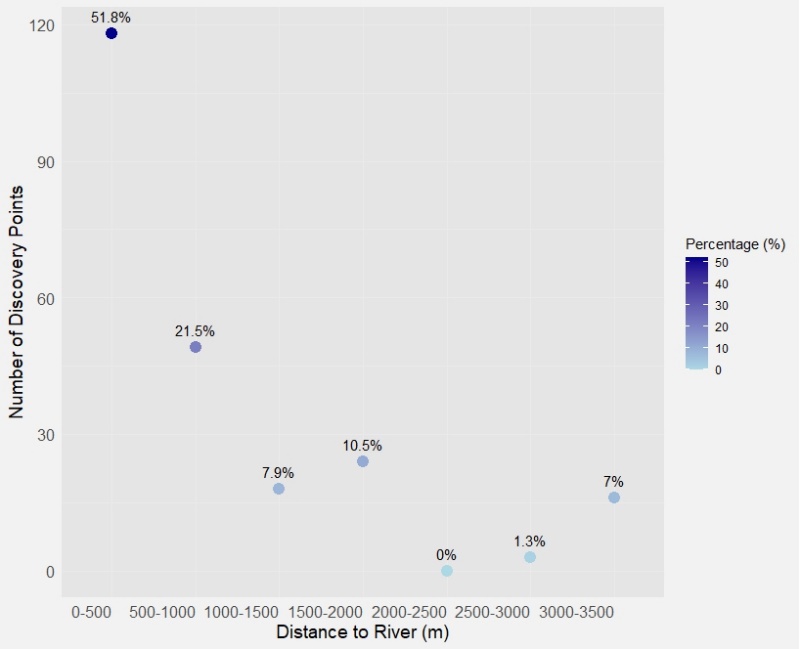

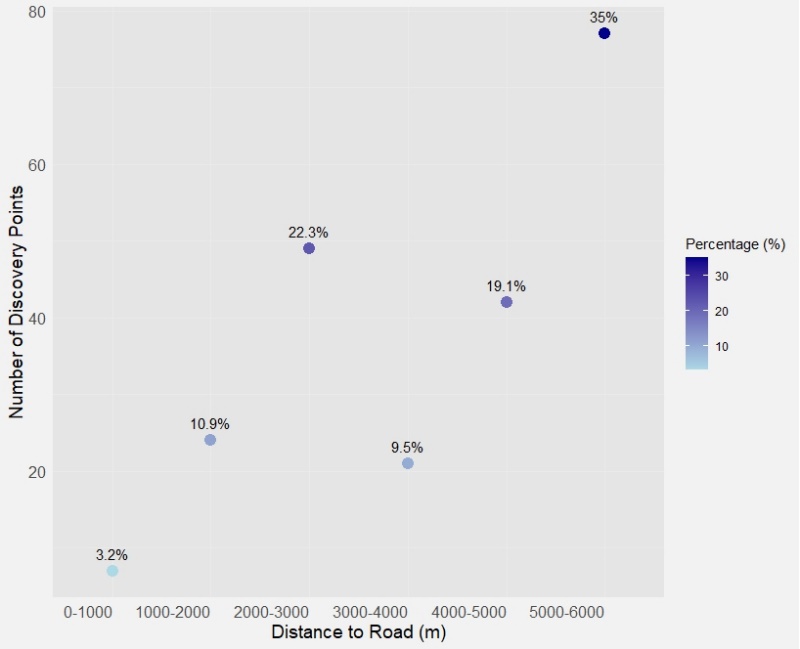


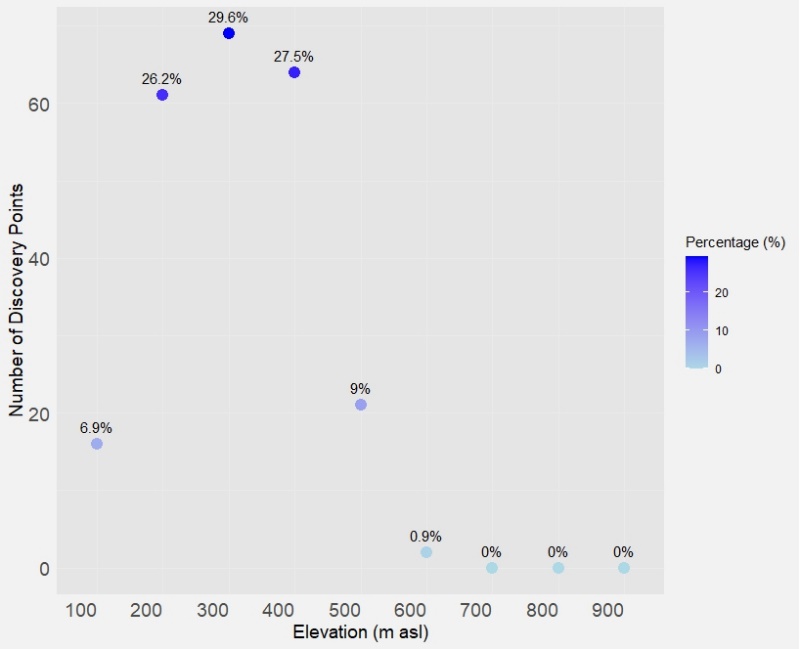

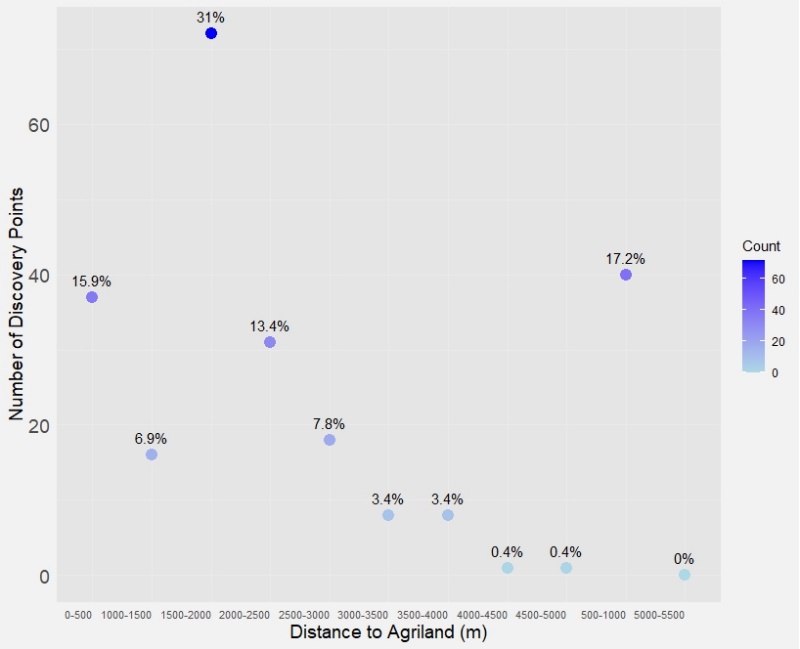

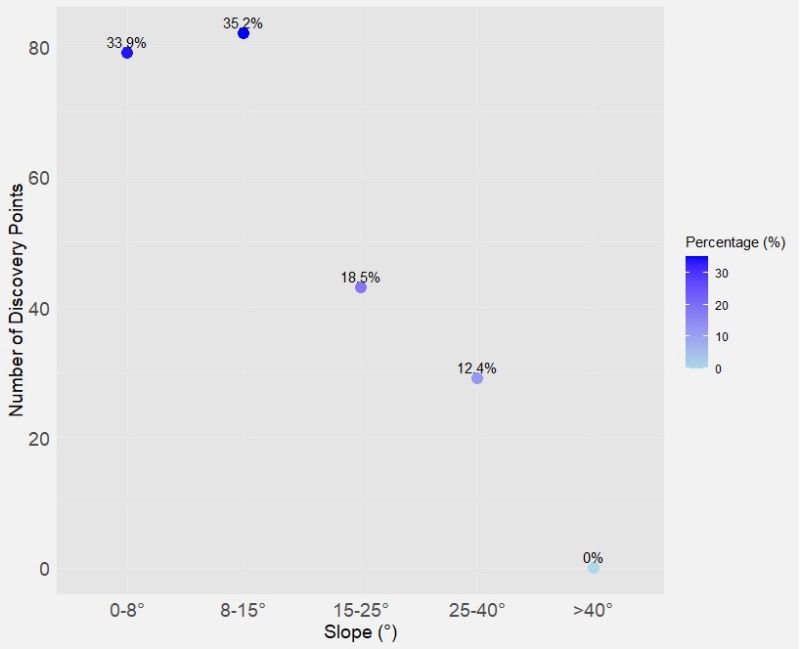

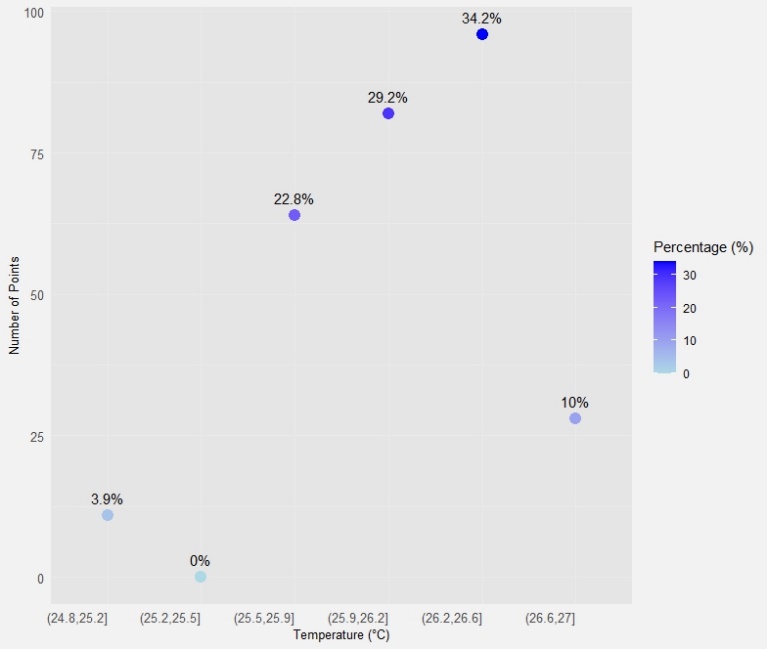

Supplement: Supplementary material 1 — Identification of the Distribution of Anoa [file bdj-13-e153431-s001.docx]
